# Morphology, input–output relations and synaptic connectivity of Cajal–Retzius cells in layer 1 of the developing neocortex of CXCR4-EGFP mice

**DOI:** 10.1007/s00429-013-0627-2

**Published:** 2013-09-12

**Authors:** Max Anstötz, Kathleen E. Cosgrove, Iris Hack, Enrico Mugnaini, Gianmaria Maccaferri, Joachim H. R. Lübke

**Affiliations:** 1 Institute of Neuroscience and Medicine INM-2, Research Centre Jülich GmbH, Leo-Brandt-Str., 52425 Jülich, Germany; 2 Department of Physiology, Northwestern University, Feinberg School of Medicine, 303 East Chicago Avenue, Chicago, IL 60611-3008 USA; 3 Department of Cell and Molecular Biology, Northwestern University, Feinberg School of Medicine, 303 East Chicago Avenue, Chicago, IL 60611-3008 USA; 4 Department of Psychiatry, Psychotherapy and Psychosomatics, RWTH/University Hospital Aachen, Pauwelstr. 30, 52074 Aachen, Germany; 5 JARA Translational Brain Medicine, Aachen, Germany

**Keywords:** CXCR4-EGFP mice, Neocortical layer 1, Cajal–Retzius cells, Input–output synapses, Long-range horizontal axons, GABAergic interneurons

## Abstract

Layer 1 (L1) neurons, in particular Cajal–Retzius (CR) cells are among the earliest generated neurons in the neocortex. However, their role and that of L1 GABAergic interneurons in the establishment of an early cortical microcircuit are still poorly understood. Thus, the morphology of whole-cell recorded and biocytin-filled CR cells was investigated in postnatal day (P) 7–11 old *CXCR4*-*EGFP mice* where CR cells can be easily identified by their fluorescent appearance. Confocal-, light- and subsequent electron microscopy was performed to investigate their developmental regulation, morphology, synaptic input–output relationships and electrophysiological properties. CR cells reached their peak in occurrence between P4 to P7 and from thereon declined to almost complete disappearance at P14 by undergoing selective cell death through apoptosis. CR cells formed a dense and long-range horizontal network in layer 1 with a remarkable high density of synaptic boutons along their axons. They received dense GABAergic and non-GABAergic synaptic input and in turn provided synaptic output preferentially with spines or shafts of terminal tuft dendrites of pyramidal neurons. Interestingly, no dye-coupling between CR cells with other cortical neurons was observed as reported for other species, however, biocytin-labeling of individual CR cells leads to co-staining of L1 end foot astrocytes. Electrophysiologically, CR cells are characterized by a high input resistance and a characteristic firing pattern. Increasing depolarizing currents lead to action potential of decreasing amplitude and increasing half width, often terminated by a depolarization block. The presence of membrane excitability, the high density of CR cells in layer 1, their long-range horizontal axonal projection together with a high density of synaptic boutons and their synaptic input–output relationship suggest that they are an integral part of an early cortical network important not only in layer 1 but also for the establishment and formation of the cortical column.

## Introduction

Cajal–Retzius cells, originally described by Ramón y Cajal (1891) and Retzius (1893, 1894) are beside so-called predecessor cells of the human embryonic forebrain (Bystron et al. 2006; for review see Bystron et al. 2008) and ‘subplate’ neurons (McConnell et al. 1989; Friauf et al. 1990; Goodman and Shatz 1993) among the earliest generated neurons in the neocortex. Since they are generated at the onset of corticogenesis around embryonic day 12–15 in *rat* and *mice*, CR cells in this species were considered as pioneer neurons (Marín-Padilla 1978; König and Marty 1981; Luskin and Shatz 1985; Bayer and Altman 1990; Hevner et al. 2003; for review see Meyer et al. 1998, 1999; Mienville 1999, but see Bystron et al. 2008). It is now believed that CR cells are born at three places: the cortical hem (Meyer et al. 2000; Takiguchi-Hayashi et al. 2004; Garcia-Moreno et al. 2007) and the septum and ventral pallidum (Bielle et al. 2005). From their place of birth they then migrate tangentially thereby covering the entire cortical mantle into the cortical pre-plate. It has been hypothesized that CR cells born at these sites populate different cortical regions and may thus play distinct, region-specific roles in neocortical development (Bielle et al. 2005). As the neocortex further develops, CR cells become, beside a heterogeneous population of GABAergic interneurons (Lavdas et al. 1999; Kubota et al. 2011; Wozny and Williams 2011; Jiang et al. 2013), the ‘principal’ neuron of the marginal zone, which will develop later to layer 1 (L1). Their laminar position is critically driven by chemokines produced by the leptomeninges via a specific signaling mediated by the *CXCR4* receptor (Paredes et al. 2006). In addition the early migration of cortical hem and septum-derived CR cells is controlled by the B cell factor (*EBF2/3*). Loss of *EBF2* in vivo causes a transient decrease in CR cell numbers in layer 1 due to a migratory defect and is accompanied by the up-regulation of *EBF3* in the cortical hem and other forebrain areas that produce CR cells. It was, therefore, suggested that *EBF2/3 *directly regulate CR cell development (Chiara et al. 2012). CR cells start to disappear in *rat* around postnatal day (P) 15 (Derer and Derer 1990; Del Rio et al. 1996, 1997; Mienville and Pesold 1999) and at P22 only <3.5 % of the population found at P3–P7 were observed in *EBF2*-*GFP mice* (Chowdhury et al. 2010).

By secreting *reelin*, an extracellular matrix molecule, CR cells have been suggested to play a key role in the structural and functional organization of the neocortex, in particular in layer formation, the inside first—outside last patterning and positioning of early and late generated principal neurons (Luskin and Shatz 1985; Noctor et al. 1999, 2001; for review see Rakic and Caviness 1995; Frotscher 1998; Marín-Padilla 1998). However, the importance of CR cells in the establishment of an early cortical network is still a subject of ongoing discussion (Derer and Derer 1990, 1992; Verney and Derer 1995; Del Rio et al. 1996, 1997; Supèr et al. 1998, 2000; Meyer et al. 2000; Perez-Garcia et al. 2001; Radnikow et al. 2002; Soda et al. 2003; for review see Frotscher 1998; Marín-Padilla 1998).

 Here, structural and functional aspects of layer 1 neurons with special emphasis to CR cells in the developing neocortex were investigated using *CXCR4*-*EGFP mice.* In these animals, CR cells are easily identifiable by their fluorescent appearance (see also *hIL*-*2/GFP mice*: Soda et al. 2003 and *Ebf2*-*GFP mice*: Chowdhury et al. 2010). We focus on their dendritic morphology and axonal projection patterns, their input–output relationship and intrinsic electrophysiology using whole-cell patch-clamp recordings combined with intracellular biocytin-filling and subsequent light- and electron microscopy. Confocal microscopy revealed that CR cells together with GABAergic interneurons form a dense network in layer 1 throughout various neocortical areas. Their density reached a peak in the first postnatal week with a marked decline until their nearly complete disappearance at P14. During the time window of their highest expression, CR cells form a dense horizontal axonal network throughout the entire layer 1 with individual neurons spanning ~1.7 mm of cortical surface.

Our morphological findings, in particular the long-range horizontal projections, the high density of synaptic boutons together with the input–output relations suggest that CR cells, beside various types of GABAergic interneurons in layer 1, are an important and integral element in an early cortical network.

## Materials and methods

All experimental procedures described in the present study were performed in accordance with the National Institute of Health (NIH) guidelines for the Care and Use of Laboratory Animals, following Northwestern University Institutional Animal Care and Use Committee (IACUC) approved protocols and complied with the guidelines laid out in the EU directive regarding the protection of animals used for experimental and scientific purposes.

### Confocal microscopy

*CXCR4*-*EGFP mice* pups aged P0–P14 were deeply anesthetized using isoflurane (3–4 % in air). The level of anesthesia was assessed by monitoring the pedal withdrawal reflex, and by pinching the tail or ear. Following deep anesthesia, mice were quickly decapitated, either immersion-fixed (P0–P4 animals) or perfusion-fixed (P6–P14) through the heart using 4 % phosphate-buffered paraformaldehyde (0.1 M PB, pH 7.4). After fixation, brains were removed from the skull and post-fixed in the same, but fresh fixative overnight at 4 °C. Brains were then cut in the horizontal plane at a thickness of 100 μm with a vibratome (Leica VT 1000, Leica Microsystems, Nussloch, Germany), collected in 0.1 M PB, counterstained with 0.1 % DAPI (Sigma Aldrich, New York, USA) diluted in 0.1 M PB, mounted on glass slides and finally embedded in Moviol (Hoechst AG, Frankfurt AM, Germany).

Laser scanning confocal images were obtained with a Nikon PCM 2000 Confocal Microscope System (Nikon, New York, USA), mounted on an eclipse microscope. Images were taken and analyzed individually or in z-stacks of different depths taken through the region of interest at different magnifications (×100 to ×400). To minimize channel spill over images were sequentially acquired and saved as ICS, IDS or TIF files. All images were further processed with Adobe Photoshop to adjust brightness/contrast without any other editing and Adobe Illustrator for high quality illustrations (Adobe Systems Inc., San Jose, CA, USA).

### Preparation of acute brain slices

*CXCR4*-*EGFP mice* (P7–P11; *n* = 32) were deeply anesthetized as described above and then decapitated. Acute slices in the horizontal plane (350–400 μm in thickness) were prepared using a Leica VT 1000 vibratome. Slices were cut in ice–cold ‘cutting’ artificial cerebrospinal fluid of the following composition (in mM): 130 NaCl, 24 NaHCO_3_, 3.5 KCl, 1.25 NaH_2_PO_4_, 1 CaCl_2_, 2 MgCl_2_, 10 glucose saturated with 95 % O_2_–5 % CO_2_ at pH 7.4. They were transferred in a storage chamber and were allowed to adjust to room temperature (20–23 °C) for 30 min prior to recordings were made. During recording, slices were continuously superfused with the extracellular solution (with CaCl_2_ increased to 2 mM and MgCl_2_ reduced to 1 mM). The intracellular pipette solution contained (in mM): 105 K methylsulfate, 10 NaCl, 20 KCl, 4 ATP-Mg, 0.3 GTP-Na_3_, 16 KHCO_3_ equilibrated with 95 % O_2_–5 % CO_2_ at pH 7.3. For morphological analysis 1–2 mg/ml biocytin (Sigma Aldrich, New York, USA) was added routinely to the internal solution.

### Visual identification of CR cells

**Fig. 1:**
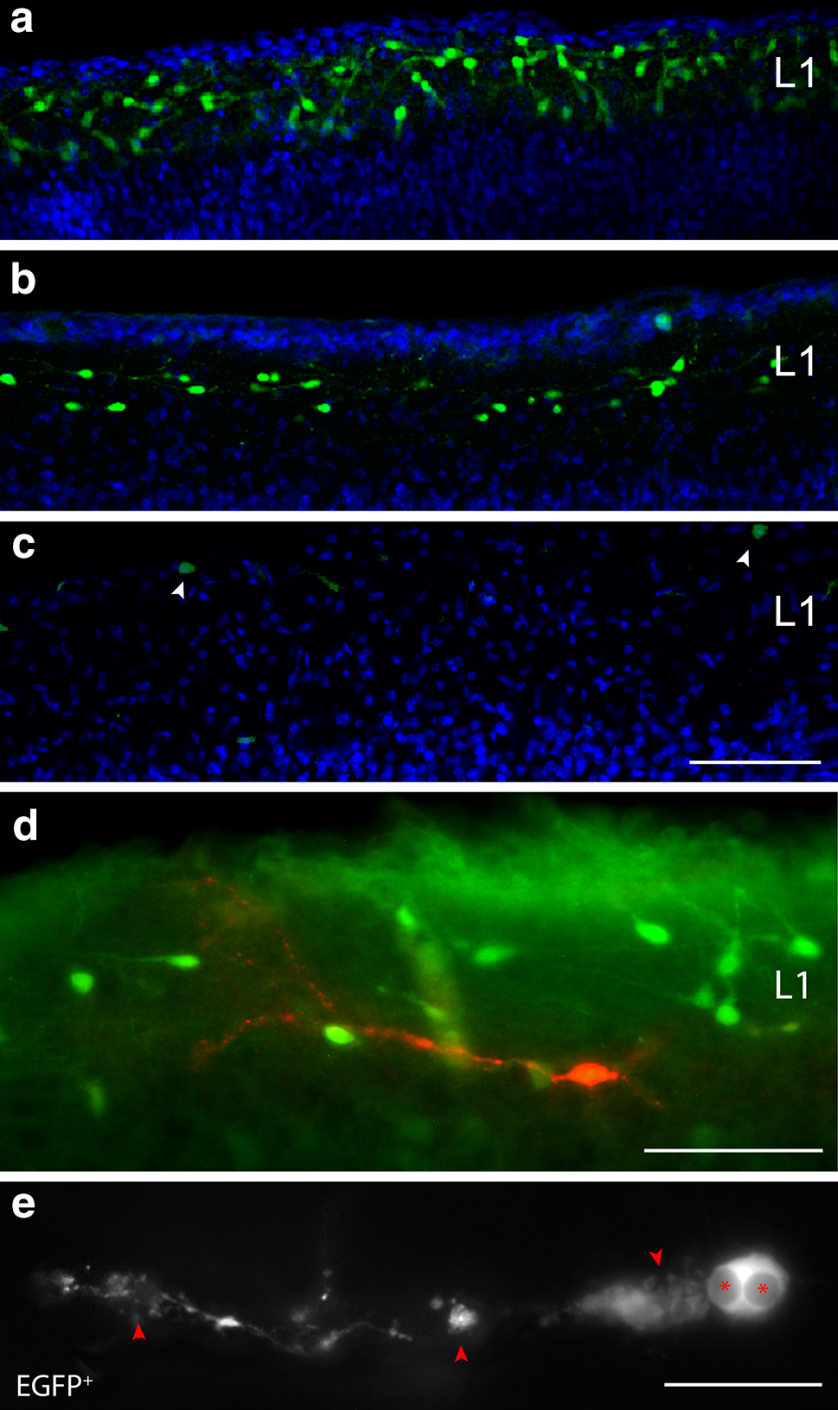
Confocal images showing the differential expression of CXCR4-EGFP in CR cells during early postnatal development and CR cells undergoing cell death. **a** CR cells in the neocortex reached their highest density and peak of expression around P4 thereby forming a dense network of neurons exclusively confined to layer 1. Note that CR cells were still present at relatively high numbers between P7 and P11 (**b**), but nearly completely disappeared at P14, with only a few scattered neurons (*arrowheads* in **c**). *Scale bar* in **a**–**c** is 100 μm. **d** Fluorescent image of a CXCR4-labeled CR cell undergoing selective cell death as revealed by Caspase-3 immunoreactivity (*red*) at the onset of apoptosis at P8. Note the numerous non-Caspase-3 positive CR cells in the surrounding neuropil in layer 1. *Scale bar* in **d** is 50 μm. **e** High power fluorescent images of a degenerating CXCR4-EGFP-labeled CR cell showing the characteristic dendritic swellings (*arrowheads*) and the pyknotic nucleus in the cell body (*asterisks*). *Scale bar* in **e** is 20 μm

Slices were placed in the recording chamber under an upright microscope (Olympus, Japan). Fluorescence of EGFP-containing CR cells was excited by an X-Cite Series 120 light source (Exfo, Ontario, Canada) and visualized using a VE1000 camera (DAGE MTI, Michigan City, IN, USA). CR cells were visually identified at ×60 magnification first by fluorescence imaging and subsequently by infrared-differential interference contrast microscopy (IR-DIC) by their location in cortical layer 1, the size and shape of their somata and the appearance of a thick stem dendrite originating from one pole of the soma (Fig. 1). All recordings and measurements were carried out at 29–31 °C.

### Electrophysiological recordings and data analysis

Pipettes were pulled from borosilicate thin glass capillaries, filled with filtered intracellular solution, with a final resistance of ~3 M*Ω*. During recording and biocytin-filling, the membrane properties and firing characteristics of CR cells were determined in the voltage- and current-clamp configuration. Recordings were carried out using a Multiclamp 700B amplifier (Molecular Devices, Sunnyvale, CA, USA). Series resistances were balanced via a bridge circuit in the current-clamp mode. Data were filtered at 3 kHz and digitized at 10–20 kHz using a Digidata A/D board and the Clampex 9 program suite (Molecular Devices, USA) and stored on a hard disk of a desktop computer. All mean values are given with the standard error.

### Histological procedures

Following recording and intracellular filling with biocytin, brain slices were fixed in 0.1 M phosphate-buffered (PB, pH 7.4) solution containing either 4 % paraformaldehyde (for light microscopy only) or 4 % paraformaldehyde and 0.5 % glutaraldehyde (for electron microscopy) at 4 °C for at least 24 h. They were then either processed for light- and/or electron microscopy as described elsewhere (Lübke et al. 2000). In brief, after incubation in ABC-Elite solution (Sigma, Aldrich, Germany) overnight, slices were pre-incubated in 3’3-diaminobenzidine (Sigma, Aldrich, Germany) and visualized by adding 0.025 % H_2_O_2_ to the solution. The reaction was stopped when dendritic and axonal processes were clearly visible. After several washing steps in 0.1 M PB sections were, after brief post-fixation in osmium tetroxide (1–2 min), either embedded in Moviol (Hoechst AG, Frankfurt AM, Germany; light microscopy) or processed for conventional electron microscopy with a longer osmification (0.5 % OsO_4_ in 0.1 M PB; 30 min) and dehydration in an ascending series of ethanol, followed by propylene oxide and final embedding in Durcopan (Fluka, Neu-Ulm, Germany). Ultrathin sections were cut using an ultramicrotome (50 ± 5 nm; Leitz Ultracut, Hamburg, Germany), counterstained with uranylacetate and lead citrate and examined with a Zeiss Libra 120 electron microscope (Zeiss, Oberkochen, Germany) using a Proscan 2 K digital camera and the SIS Analysis software (Olympus Soft Imaging System, Münster, Germany).

### Morphological reconstructions of biocytin-filled neurons

Only neurons for which a complete physiological analysis was made and that had no obvious truncation of their dendritic and axonal profiles were used for qualitative and quantitative analysis of their morphology. Neurons were photographed at various magnifications (Olympus BX61 microscope equipped with the SIS analysis software) to document their dendritic morphology and axonal projection. Representative examples were reconstructed using the *Neurolucida* software (MicroBrightfield Europe, Magdeburg, Germany) equipped to an Olympus BX61 microscope (Olympus, Hamburg, Germany). These reconstructions provided the basis for further quantitative morphological analysis of the following parameters: (1) total length of axonal collaterals, (2) maximal horizontal field span of axonal collaterals, (3) mean length and number of axonal collaterals (segments), (4) axonal branch points, (5) total number and density of light microscopically identified synaptic boutons, (6) mean length of the dendritic tree, (7) mean length and number of dendritic side branches (segments), (8) dendritic branch points and (9) soma diameter. Measurements were not corrected for shrinkage. For all data, mean ± SD and the median were calculated.

### GABA postembedding immunogold labeling

The immunogold staining procedure was carried out as described by Somogyi et al. (1985), using a commercially available antiserum against GABA (Sigma, München, Germany). The immunostaining was carried out on droplets of Millipore-filtered solutions in humid Petri dishes. Immersion in 1 % periodate (10 min) was followed by several washes in double-distilled water. Thereafter, the grids were transferred through 2 or 5 % sodium metaperiodate (10–30 min) and rinsed several times in double-distilled water and three times in Tris-buffered saline (TBS, pH 7.4). After pre-incubation in 1 % ovalbumin dissolved in TBS (30 min), grids were incubated in rabbit anti-GABA antiserum (1:5,000, in 1 % normal goat serum in TBS). After rinsing in TBS and 50 mM Tris buffer (pH 7.4) containing 1 % bovine serum and 0.5 % Tween 20 (10 min), grids were incubated in the secondary antibody (goat anti-rabbit IgG-coated colloidal gold, 10 nm) for 2 h (diluted 1:10, in darkness). After rinsing in 2 % glutaraldehyde (10 min), grids were washed again in double-distilled water and stained with uranyl acetate and lead citrate. In control experiments without the primary antibody and sections processed for GABA post immunogold labeling almost no or only low background labeling was detected, whereas labeling of GABAergic structures clearly exceeded the mean gold particle density of the maximum background staining (by at least 4 standard deviations). Ultrathin sections were examined using a Zeiss Libra 120 electron microscope (Zeiss, Oberkochen, Germany) equipped with a Proscan 2 K bottom-mounted camera and the SIS analysis software (Olympus Soft Imaging System, Münster, Germany).

### Caspase-3 immunohistochemistry

 In order to detect apoptotic CR cells, immunohistochemistry was carried out using a commercially available antiserum against Caspase-3 (anti-Caspase-3, Cell Signaling, Frankfurt AM, Germany) on *CXCR4-EGFP mice* ranging in age between P4-P12. 50 μm thick sections were cut in the horizontal plane using a Leica VT1000 vibratome and collected in 0.1 M phosphate-buffered saline (PBS). The immunostaining was done on free-floating sections. After several brief rinses in 1× tris-buffered saline containing 1 % Triton-X 100 (TBST), sections were transferred into 70 % formic acid (diluted in double-distilled water for 5 min) and subsequently washed three times in 1 × TBST. Then sections were pre-incubated in 0.1 M PBS containing 10 % NGS/0.5 % Triton X-100 for 1 h. After several brief washing steps sections were transferred into anti-caspase-3 (1:400) diluted in 1 % BSA/TBST overnight at 4 °C while shaking. After several brief washing steps in 1 × TBST (1 min each) sections were incubated in the secondary antibody Alexa 568 gt α rb (1:800; Invitrogen, Darmstadt, Germany) diluted in 1 % BSA/TBST for 2 h in the dark. Finally, sections were washed several times in 1 % TBST, counterstained in DAPI (1:10,000 diluted in PBS), mounted on glass slides and cover slipped. Sections were viewed using an Olympus BX61 microscope equipped with the appropriate fluorescence filters and documented using the SIS software (SIS Olympus, Münster, Germany).

### Axonal and dendritic polar plots and density maps

Individual polar plots were generated from the *Neurolucida* reconstructions of individual CR cells using the *Neuroexplorer *software (MicroBrightfield Europe, Magdeburg, Germany). From these plots a normalized average polar plot was obtained for the dendritic and axonal domains.

In addition, two-dimensional (2D) maps of axonal and dendritic ‘length density’ were constructed using the computerized 2D reconstructions (for details see Lübke et al. 2003). First all reconstructed CR cells were projected in a 2D plane, centered to the axonal initial segment and then measured in a 50 × 50 μm cartesian grid, yielding a raw density map. Continuous 2D density functions were constructed using bicubic interpolation in Mathematica 7 (Wolfram Research, Champaign, IL, USA). Both, dendritic and axonal density maps were merged together. Furthermore, care was taken to minimize shrinkage in the *x*–*y* plane using water-based embedding media (see above).

## Results

### Selective cell death through apoptosis of neocortical CR cells

It is still controversially discussed also with respect to different species when CR cells in the neocortex reached their peak of postnatal expression, when they begin to disappear from layer 1 and undergo selective cell death by apoptosis or whether they are diluted in the volume of the growing neocortex. In *rat*, the process of CR cell loss starts around P15 (Derer and Derer 1990; Del Rio et al. 1996, 1997; Mienville and Pesold 1999). Using confocal microscopy in *CXCR4*-*EGFP*-expressing *mice* (Cosgrove and Maccaferri 2012), CR cells are easily identifiable by their fluorescent appearance. Their density and distribution pattern was investigated in a time window between P0 to P14. Between P3 to P7 CR cells displayed the highest density throughout layer 1 (Fig. 1a). Interestingly, differences in their density and distribution pattern were found between individual cortical regions, for example the motor, somatosensory, visual and piriform cortex with the highest density in layer 1 of the entorhinal and somatosensory cortex. *CXCR4*-*EGFP*-labeled CR cells occupied the entire volume of layer 1 from the pial surface to the layer 1–layer 2/3 border (Fig. 1a). From the beginning of the second postnatal week (P8) the density of CR cells declined progressively (Fig. 1b, c). CR cells undergo selective cell death through apoptosis as shown by Caspase-3 immunohistochemistry (Fig. 1d) and signs of degeneration such as dendritic swellings and pyknotic nuclei in cell bodies (Fig. 1d, e), although the EGFP-labeling is still detectable until their disappearance (Fig 1e). At P14 only a few scattered neurons in layer 1 were found (Fig. 1c). This sharp decline was observed in all neocortical regions and ages although interregional differences were observed.

Strikingly, in the hippocampal formation CR cells were still present at very high densities especially in the inner and at the border of the inner/outer molecular layer of the dentate gyrus and the stratum lacunosum moleculare. In *CXCR4*-*EGFP mice,* neocortical CR cells seem to reach their peak in maturity and expression around P7–P11 (personal observation), therefore, all patch-clamp recordings were performed within this time window.

### Morphology of CR cells in the neocortex of CXCR4-EGFP mice

We never encountered EGFP-positive cells in layer I that were not CR cells since all biocytin-fillings of fluorescent-labeled neurons displayed CR cell morphologies. We never recorded from EGFP-labeled neurons that afterwards turned out to be GABAergic interneurons.

**Fig. 2:**
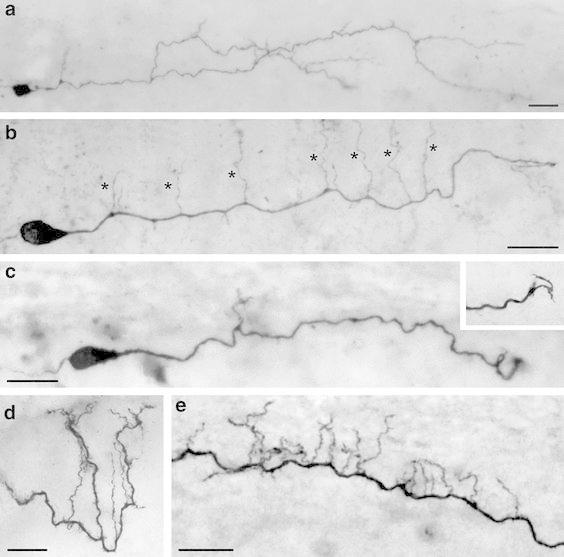
Dendritic morphology of neocortical CR cells. **a**–**c** Light microscopic images of three biocytin-filled CR cells from P11 old animals showing the heterogeneity in their dendritic morphology with respect to the length of the stem dendrite, side branches and frequency of spine-like, filopodial appendages. The CR cell in **a** shows a relatively high degree of collateralization and number of side branches covered with spine-like, filopodial appendages. The CR cell in **b** possessed a single thick stem dendrite with vertically oriented side branches (marked by *asterisks*); whereas the CR cell in **c** is an example with a short and smooth stem dendrite with only a single side branch. *Inset:* On dendritic side branches sometimes growth cone-like structures were observed. **d** High power photomicrograph of a vertically oriented tuft dendrite with side branches terminating near the pial surface. **e** Example of a CR cell stem dendrite with a high degree of short vertically, pial-oriented side branches. All figures are oriented such that the pial surface is on top. *Scale bars* in **a**–**e** are 20 μm

Biocytin-filled CR cells were found throughout the entire layer 1 (Figs. 1, 2, 4, 5). However, the majority was located in the middle portion of layer 1 and only a few CR cells were located near the pial surface or at the layer 1/layer 2/3 border (Figs. 4, 5a1, a2). Most CR cells were identified under fluorescent and differential-interference contrast microscopy by the size and shape of their somata, a prominent mainly horizontally oriented stem dendrite originating from one pole of the soma and by their characteristic action potential firing pattern (Zhou and Hablitz 1996a, b; Hestrin and Armstrong 1996; Kilb and Luhmann 2000; Radnikow et al. 2002; this study). Like in *rats* (Radnikow et al. 2002) CR cells in *CXCR4*-*EGFP mice* can be subdivided in typical and ‘atypical’ CR cells according to their dendritic arborization as well as their axonal projection pattern (Figs. 4, 5).

### Typical CR cells

Typical CR cells represent the majority (~80 % of the total population; *n* = 75) of these neurons in layer 1 (Fig. 2a–c). Typical CR cells had ovoid or elongated somata with a horizontal diameter of 15.21 ± 2.77 μm (see also Table 1) and were characterized by a prominent horizontally oriented thick stem dendrite originating from one pole of the soma (Figs. 2a–c, 5a1, a2). Together with the axon they show the typical bipolar orientation characteristic for these neurons (Fig. 7a1, a2). The stem dendrite gives rise to several secondary and tertiary dendrites of various length (20–100 μm) that were mainly oriented vertically (Fig. 2b, e) towards the pial surface where they terminate. Interestingly, some distal dendrites were even seen to enter the ependym above the pial surface (Fig. 2d). The majority of typical CR cell dendrites were covered with spine-like, filopodial protrusions (Fig. 2c–e). However, some neurons also had smooth dendrites without any appendages and only short dendrites (Fig. 2c). Occasionally, typical CR cells formed a complex, sometimes vertically oriented terminal tuft at the most distal portion of the stem dendrite (Fig. 2d). Some of the stem dendrites could be followed over wide distances with a mean dendritic length of 557.1 ± 275.0 μm (minimum 159.7 μm, maximum 941.7 μm; see also Table 1). A few dendrites terminated in growth cone-like endings (Fig. 2c inset) that could be found even on CR cells recorded and biocytin-filled in P11 old *CXCR4*-*EGFP mice* suggesting that these neurons were still in the process of dendritic maturation. However, the arrangement and orientation of the dendritic tree of typical CR cells was always strictly unipolar with a small vertical dimension of their dendrites as indicated by the averaged polar plots (Fig. 7a2).

**Table 1:** Quantitative parameters of the somatodendritic and axonal domains of CR cells in layer 1

Neuron reference number	Total length of axonal collaterals (μm)	Maximal field span of axonal collaterals (μm)	Mean length of axonal segments (μm)	Axonal nodes	Number of synaptic boutons	Boutons/100 μm axonal segment	Total length of the dendritic tree (μm)	Mean length of dendritic segments (μm)	Dendritic nodes	Soma diameter (μm)	CR cell type
1_23910A	3009.3	898.4	61.4	24	987.7	32.8	746.1	75.6	16	13.5	Typical
6_101012E1	2830.5	818.0	149.0	26	836.9	29.6	761.9	54.4	14	15.2	Typical
6_101012E2	533.0	170.8	66.6	20	190.8	35.8	720.4	45.0	7	16.9	Typical
8_101018A, B	4454.1	1006.5	262.0	22	1156.2	26.0	472.9	59.1	9	22.4	Typical
9_101021B, C	4106.6	1043.4	257.7	23	1138.7	27.7	840.6	64.7	23	13.4	Typical
10_101021F1	1629.5	621.2	162.9	10	516.0	31.7	355.4	35.5	9	15.6	Typical
10_101021F2	2012.5	577.9	287.5	11	684.2	34.0	774.5	19.6	40	15.5	Typical
14_101130C	2286.6	624.1	285.8	15	701.3	30.7	159.7	79.9	1	15.3	Typical
19_101209D	2770.6	1313.8	277.1	15	930.5	33.6	923.8	92.4	10	10.6	Typical
19_101209F	5626.7	1655.3	312.1	30	1573.2	28.0	290.8	48.5	7	14.4	Typical
20_101210C	3123.3	739.0	347.0	12	1046.8	33.5	941.7	72.4	33	14.6	Typical
23_101215C	5193.3	1185.3	399.5	28	1501.6	28.9	180.4	36.1	4	15.8	Typical
25_101220C, D, E	2928.5	920.2	225.3	22	705.7	24.1	248.4	49.7	5	11.4	Typical
26_101220F	3147.6	893.6	286.1	15	706.7	22.5	382.1	42.5	9	18.3	Typical
Mean	3118.0	890.5	241.4	19.5	905.5	29.7	557.1	55.4	13.4	15.2	Typical
SD	1320.9	346.0	95.3	6.3	356.9	3.8	275.0	19.2	10.9	2.8	Typical
Median	3009.3	893.6	262.0	20.0	905.5	30.2	557.1	54.4	9.0	15.2	Typical
3_23910C2	1814.9	484.1	302.5	10	417.9	23.0	491.6	35.1	18	12.2	Atypical
11_101022B	1844.1	495.1	263.4	8	263.5	14.3	565.9	47.2	9	16.3	Atypical
12_101022D	3604.9	1222.9	600.8	12	1264.7	35.1	490.1	81.6	6	12.9	Atypical
13_101119B	4984.9	873.2	453.2	15	1253.1	25.1	743.7	46.5	29	14.3	Atypical
16_101201F	2493.6	686.1	249.4	10	540.1	21.7	492.7	54.8	9	17.3	Atypical
17_101202C	2526.2	829.9	421.1	6	784.7	31.1	415.5	69.3	6	12.7	Atypical
18_101208G	4387.2	1041.8	398.8	16	1252.1	28.5	863.9	45.5	32	11.2	Atypical
22_101215A	5216.9	1156.0	320.0	17	1527.2	29.3	949.1	39.9	14	21.3	Atypical
23_101215D	2595.0	697.1	370.7	9	758.0	29.2	988.0	89.8	15	13.8	Atypical
Mean	3274.2	831.8	375.5	11.4	895.7	26.4	666.7	56.6	15.3	14.7	Atypical
SD	1240.7	253.3	103.4	3.6	418.4	5.8	20.0	18.1	9.0	3.0	Atypical
Median	2595.0	829.9	370.7	10.0	784.7	28.5	565.9	47.2	14.0	13.8	Atypical
Significant difference			*	*							All
Mean	3179.1	867.6	293.9	16.3	901.6	28.5	600.0	55.9	14.1	15.0	All
SD	1292.3	314.3	118.3	6.7	382.2	5.0	256.9	18.8	10.2	2.9	All
Median	2928.5	873.2	286.1	15.0	836.9	29.2	565.9	49.7	9.0	14.6	All

To demonstrate variability of morphological parameters between typical and ‘atypical’ CR cells data from individual neurons are listed

Significant value *p* < 0.01

**Fig. 3:**
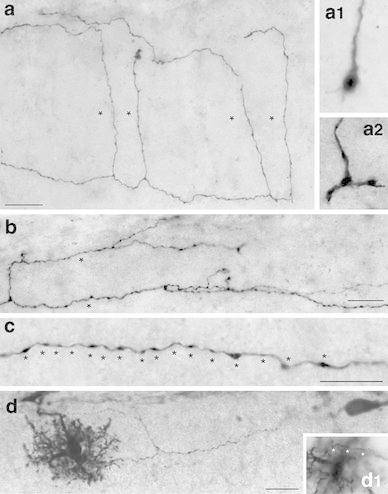
Axonal features of neocortical CR cells. **a** Axonal projection pattern of a typical CR cell with long vertically oriented axonal collaterals (marked by *asterisks*) spanning the entire volume between the pial surface and the layer 1/layer 2/3 border. Some of these collaterals still bear growth cones at their tips (**a1**, **a2**). **b** Axonal projection pattern of a typical CR cell with long horizontally oriented axonal collaterals, often running parallel to the main axon and the pial surface. **c** High power photomicrograph showing the high density and distribution of synaptic boutons (marked by *asterisks*) along a single axonal collateral. Note the different size of the synaptic boutons. **d** Dye- and synaptic coupling between a CR cell and a neighboring end foot astrocyte. Biocytin-filling of a single CR cell often led to co-labeling of astrocytes, but not of other L1 neurons or neurons in the underlying cortical layers 2/3 to 6. Note the invasion and ramification of the CR cell axon marked by *asterisks* into the astrocytic tree (**d1**). *Scale bars* in **a**, **b**, **d** are 20 μm and in **c** 10 μm

**Fig. 4:**
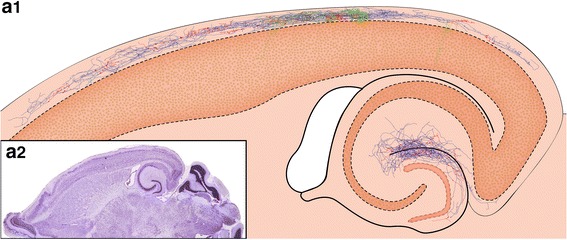
Location and distribution of all biocytin-filled and reconstructed CR cells and GABAergic interneurons in layer 1 of the neocortex. **a1** *Neurolucida* reconstructions of all investigated and reconstructed CR cells and GABAergic interneurons in layer 1 of the neocortex. The somatodendritic domain of CR cells is given in *red*, their axonal arborization in *blue*. The somata and dendrites of the GABAergic interneurons are depicted in *orange*, their axons in *green*. CR cells formed a dense, horizontal network exclusively confined to layer 1 with individual CR cells spanning ~1.7 mm of cortical surface whereas most GABAergic interneurons form a more local plexus in layer 1 or project to the underlying cortical layers. In contrast, CR cells located in the dentate gyrus or str. lacunosum moleculare of the hippocampus always had two axonal domains, a dense local and a projection domain to various subregions of the hippocampus and even to the entorhinal cortex. **a2** Nissl-stained section showing the plane of sectioning of the acute slices. For electrophysiological recordings slices were used in which the ‘barrel field’ of the somatosensory cortex and the hippocampal formation were visible

**Fig. 5:**
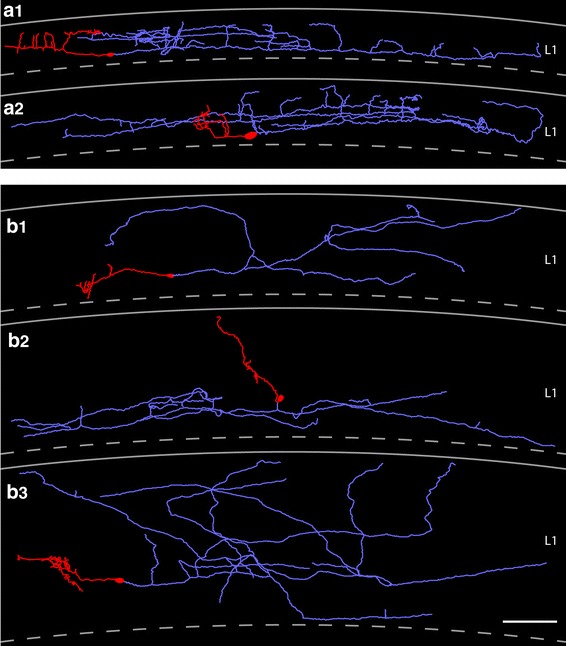
*Neurolucida* reconstructions of typical and ‘atypical’ CR cells in layer 1 of the neocortex. **a1**, **a2** Two representative examples of CR cells showing the typical dendritic configuration and axonal arborization characteristic for CR cells. The somatodendritic domain is given in *red*, the axonal arborization in *light blue*. Top most *lines* indicate the pial surface, *dashed lines* the border between layer 1 and layer 2/3. **b1**–**b3** Three representative examples of ‘atypical’ CR cells. These CR cells either showed alterations in their somatodendritic orientation and arborization (**b2**) or had a much broader axonal field spanning nearly the entire volume of layer 1 (**b1**, **b3**). Some *color code* as in **a1** and **a2**. *Scale bar* in **a1**–**b3** is 100 μm

The main axon emerged nearly always from the opposite pole of the soma and in two neurons from the thick stem dendrite. The majority (~70 %) of typical CR cells investigated possess an axon that projects over a wide range of cortical surface with individual long-range horizontal collaterals spanning ~1.7 mm thereby forming a dense network in layer 1 (Figs. 4, 5a1, a2, 7a1, c, see also Table 1). These long-range horizontal collaterals were seen to run parallel to the pial surface (Fig. 3b) or even the ependym with a mean maximum field span of 890.53 ± 346.01 μm (minimum 170.8 μm, maximum 1655.3 μm; *n* = 14). On its course the main axon gives rise to several vertically (Fig. 3a) or slightly horizontally oriented side branches of various length, often running parallel to the main axon forming a dense network of axonal collaterals (Figs. 3b, 4, 5, 7a1, c). Occasionally, some of these collaterals formed clusters in layer 1 (Fig. 5a1, a2). Interestingly, on ~30 % of the axonal collaterals growth cones were observed (Fig. 3a1, a2) indicating that these axons were still in the process of elongation.

**Fig. 6:**
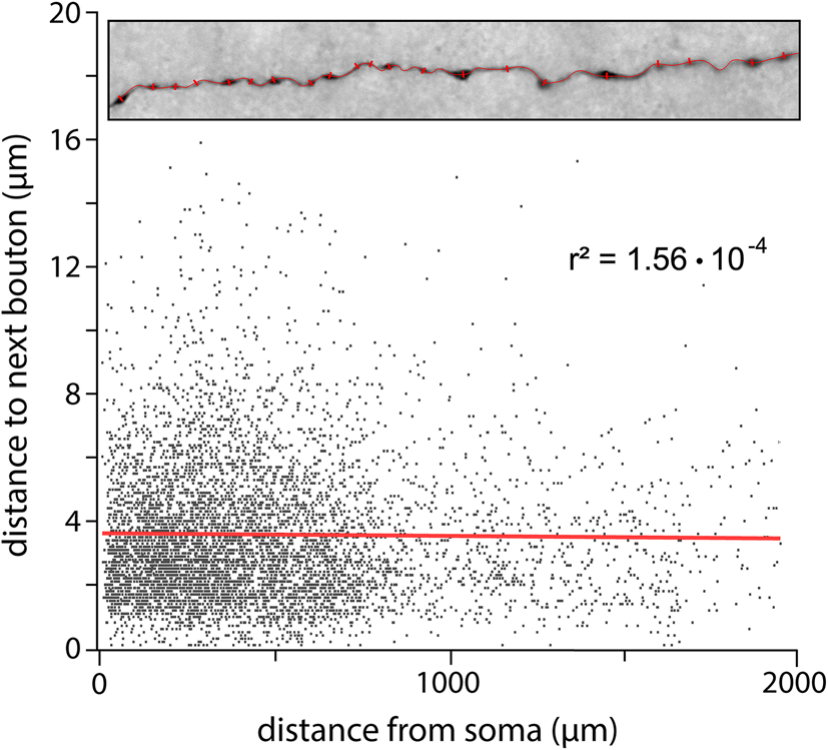
Quantitative analysis of the axonal bouton density and distribution. *Dot plot* showing the density, distribution and distance of synaptic boutons along the reconstructed axons of CR cells. *Inset* axonal collateral of a CR cell showing the method of analysis. First, a *line* was drawn along the axon; then a marker was placed at the center of each synaptic bouton which allows the interpolation of the distance between two adjacent boutons. Note that the ‘distance to next bouton’ seems to be independent from the distance to the soma as shown by the *red fitting line* and the *r*² value

**Fig. 7:**
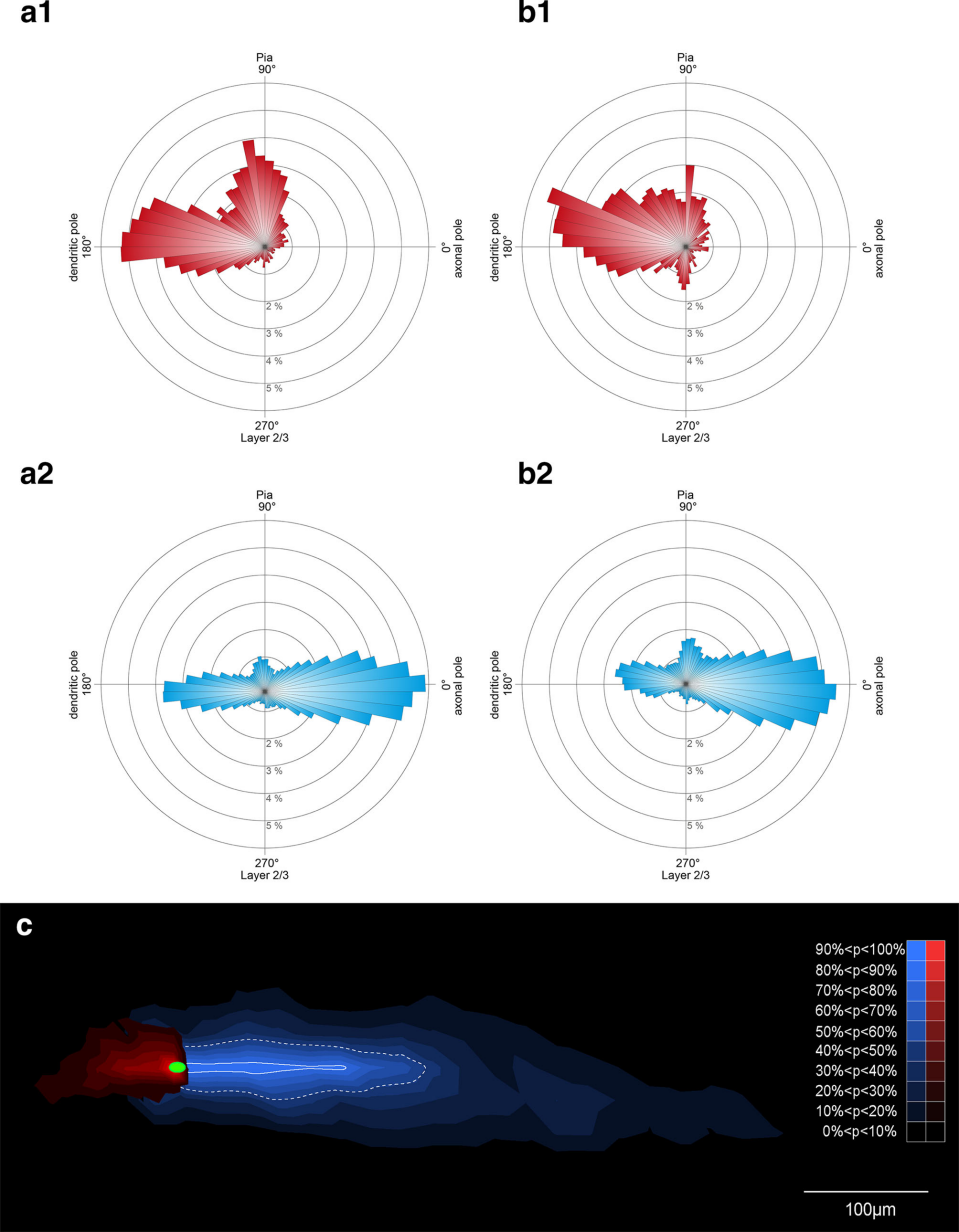
Quantitative analysis of the dendritic tree and axon of CR cells. Averaged and normalized polar plots showing the distribution pattern of the dendritic and axonal domains of typical (**a1**, **a2**) and ‘atypical’ (**b1**, **b2**) neocortical CR cells. The dendritic domain is given in *red*, the axonal domain in *blue*. **b1**–**b2** Note the high degree of polarity of both dendrites and axons but the slight difference in the orientation and distribution of dendrites and axons between typical and ‘atypical’ CR cells. **c** Averaged and normalized density plot showing the extension of the axonal domain (*blue*) and  the dendritic arborization (*red*) of all neocortical CR cells investigated. The solid *white line* represents 50 % and the *dashed line* 80 % probability of appearance in the distribution of the axon

Another striking feature of CR cells was the comparably high density of synaptic boutons (Figs. 3c, 6; see also Table 1). On average 28.01 synaptic boutons per 100 μm were found. Interestingly, no significant difference was found for proximal and distal portions of the axon (Fig. 6) although the density of axonal collaterals progressively decreased. Thus, the high number of axonal boutons together with the long-range axonal collaterals suggests a high degree of connectivity of these neurons (Figs. 4, 7c) with other CR cells or L1 GABAergic interneurons (see "Discussion").

### ‘Atypical’ CR cells

The other subpopulation of CR cells differed from typical CR cells with respect to their dendritic configuration and/or the shape, density and projections of their axons (Fig. 5b1–b3, see also Table 1). They were classified as ‘atypical’ CR cells (see also Radnikow et al. 2002). However, the averaged difference in the orientation and extent of their dendritic and axonal field was vertically only slightly broader when compared with that of typical CR cells (compare Fig. 7a1, a2 with b1, b2). They were fewer in number and their location in layer 1 was somewhat different from those of typical CR cells.

The somata of ‘atypical’ CR cells (horizontal diameter 14.6 ± 2.9 μm, see Table 1) were also found throughout the entire layer 1 (Fig. 5b1–b3) although several neurons were located directly underneath the pial surface near the ependym. However, their dendritic arborization was diverse when compared with typical CR cells. ‘Atypical’ CR cells usually also possess a thick stem dendrite that originates directly from the soma and then projects vertically or slightly horizontally within layer 1 (Fig. 5b1). Sometimes the stem dendrites give rise to twinned dendrites or had shorter secondary and higher order dendrites that branched off and then took a vertical and/or horizontal course. In a few cases even somata and proximal dendrites were vertically oriented (Fig. 5b2). In contrast to typical CR cells, the majority of dendrites were smooth, spine-like appendages were only rarely observed. Some neurons formed small terminal tufts at their tips (Fig. 5b1, b3). In summary, the dendritic configuration of ‘atypical’ CR cells is more heterogeneous (compare Fig. 5b2 with a2).

For most CR cells with an ‘atypical’ dendritic configuration the axonal projection and density of synaptic boutons was nearly similar to that of typical CR cells with a maximal field span of ~1.2 mm (compare Fig. 5b1–b3 with a1, a2, see Table 1). Although the axonal plexus of ‘atypical’ CR cells was broader sometimes spanning the entire volume of layer 1 (Fig. 5b1, b3), the axon was also located and confined to layer 1 (but see Radnikow et al. 2002). Furthermore, the axonal plexus of some neurons was less dense and less collateralized than that of typical CR cells (compare Fig. 5a1, a2 with b1–b3). Interestingly, no significant difference in the density of synaptic boutons was observed between typical and ‘atypical’ CR cells.

### Phenomenon of dye-coupling

**Fig. 8:**
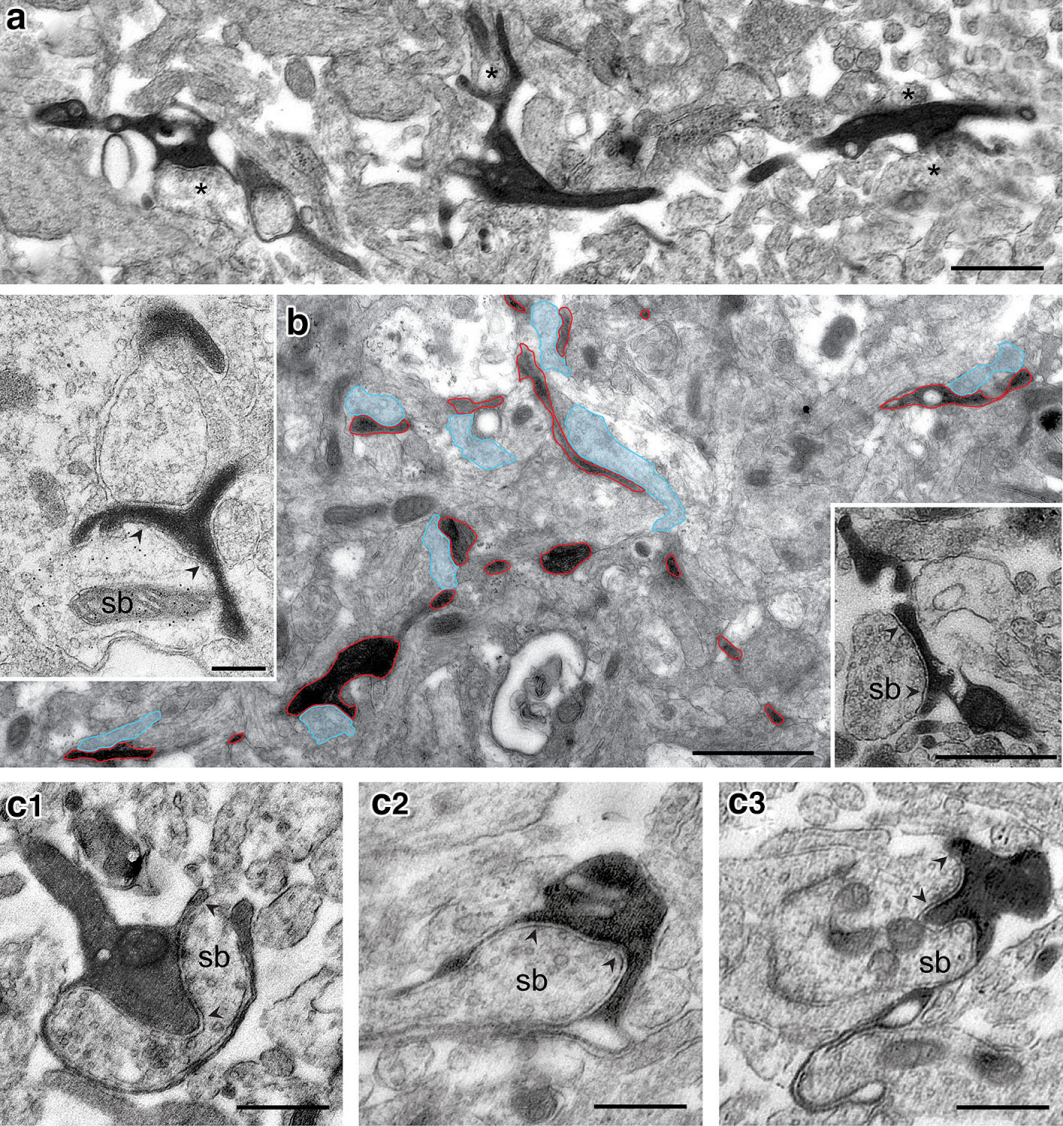
Synaptic input to CR cells. **a** Low power electron micrograph of the initial segment of a stem dendrite near the somatic region of a CR cell filled with biocytin (as indicated by the dark DAB-reaction product). CR cells receive relatively dense synaptic input on thin dendritic spine-like appendages (middle dendritic segment) and dendritic shafts (left and right dendritic segment) as marked by the *asterisks*. Note the large extracellular space within the neuropil that is characteristic for this age (P10). *Scale bar* in **a** is 1 μm. **b** Low power electron micrograph of a terminal tuft (*red contours*) terminating close to the pial surface. Note that the dendritic segments are densely targeted by synapses highlighted in light transparent *blue*. *Insets:* High magnification of two dendritic spine-like appendages receiving GABAergic (*left* *inset*) and non-GABAergic (*right inset*) input from synaptic boutons (*sb*). Note the appearance of so-called ‘docked’ synaptic vesicles at the presynaptic density (marked by *arrowheads*) as an indicator for a functional synapse. **c1**–**c2** Two typical examples of input synapses (*sb*) on a small caliber proximal (**c1**) and distal (**c2**) spine-like appendage. The presynaptic densities are marked by *arrowheads*. Note the fine filopodial processes closely ensheathing the synaptic bouton and the occurrence of ‘docked vesicles’ at the presynaptic density. **c3** Putative fine glial process in close apposition to a synaptic bouton (*sb*). The presynaptic density is also marked by *arrowheads*. Note the row of ‘docked’ vesicles along the presynaptic membrane suggesting a synaptic bouton-astrocytic contact site. *Scale bars* in **c1**–**c3** are 0.25 μm

Another striking feature in *CXCR4*-*EGFP mice* was that no dye-coupling between CR cells, GABAergic interneurons in layer 1 or neurons in the underlying cortical layers was observed (see "Discussion"). Labeling of a single CR cell or a GABAergic interneuron did not result in co-labeling of adjacent L1 neurons or the underlying cortical layers. However, in 30 % of our cases biocytin-filling of a single CR cell lead to a co-staining of so-called end foot astrocyte (Fig. 3d). Furthermore some axonal collaterals were seen to invade the astrocytic tree (Fig. 3d inset) suggesting that dye-coupling or even putative synaptic coupling exists in some CR cells and point at least partially against the non-existence of dye-coupling in *CXCR4*-*EGFP mice*. Electron microscopy revealed that, in cases where an end foot astrocyte was co-filled, DAB-labeled small caliber, putative astrocytic processes, were found near the pial surface in close contact to unlabeled synaptic boutons (Fig. 8c3) suggesting a synaptic connection between the two structures. However, since DAB-labeled distal dendritic segments of CR cells also show fine filopodial processes at the tips of spine-like appendages closely ensheathing synaptic boutons (compare Fig. 8c1, c2 with c3), an unequivocal decision cannot be made on the ultrastructural features alone.

### Input synapses to CR cells

Somatic and dendritic input synapses have been found on embryonic CR cells (König and Marty 1981), however, only a few scattered synapses were found postnatally (König and Marty 1981; Derer and Derer 1990, 1992) although in P10 old* rats *GABAergic and glutamatergic, namely NMDA inputs were found on both somata and dendrites, respectively (Radnikow et al. 2002). Furthermore, electrophysiological (Kilb and Luhmann 2001; Soda et al. 2003; Cosgrove and Maccaferri 2012) and imaging studies in rodents also suggest that CR cells may receive glutamatergic, GABAergic, serotonergic and noradrenergic inputs (Kim et al. 1995; Schwartz et al. 1998; Aguiló et al. 1999; Kilb and Luhmann 2001).

We therefore looked for synaptic inputs on morphologically and physiologically identified CR cells using a combination of intracellular biocytin-labeling and GABA postembedding immunohistochemistry (*n* = 3 CR cells). In contrast to earlier studies, but in agreement with findings in the *rat* (Radnikow et al. 2002), CR cells receive relatively dense synaptic input from non-GABAergic and to a much lesser extent from GABAergic synapses (Fig. 8b left and right insets). Synaptic boutons terminating onto CR cells were found on somata (not shown), proximal (Fig. 8a) and distal dendrites (Fig. 8b) directly on dendritic shafts (~70 % of the total of synapses investigated; Fig. 8a, b left and right dendritic segment) or on spine-like appendages (~20 % of the total of synapses investigated; Fig. 8c2, c3). The remaining GABAergic input was found particularly at the somatic region (not shown) or shaft dendrites of different caliber (Fig. 8b left inset) presumably from GABAergic interneurons in layer 1 (see "Discussion"). Interestingly, non-GABAergic inputs, presumably glutamatergic inputs were frequently observed at relatively high numbers even on terminal tuft dendrites terminating near the pial surface (Fig. 8b, c2, c3).

The origin of the non-GABAergic input is still rather unknown. However, the axonal projection and density of CR cells (see Fig. 4) suggest that the majority of these inputs come from other CR cells. Another source of putative non-GABAergic synaptic input may arise from ascending axons from glutamatergic neurons, probably pyramidal cells in different cortical layers and layer 4 excitatory spiny neurons.

### Postsynaptic target structures of CR cells

**Fig. 9:**
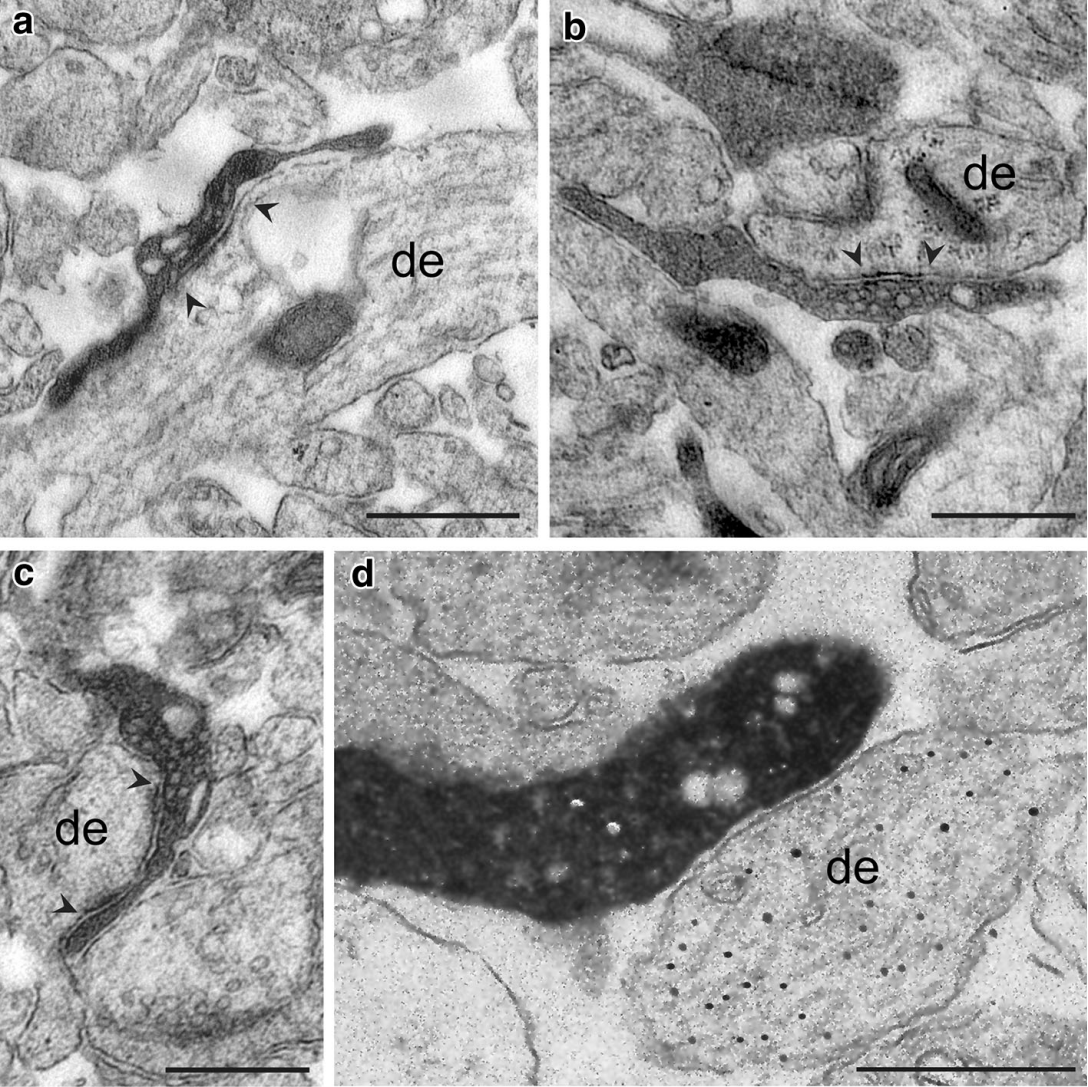
Synaptic output of CR cells. **a**–**c** Synaptic contacts established by the CR cell axonal collaterals (dark DAB-labeled structures) on postsynaptic target structures. *En passant* synaptic boutons are located on dendritic shafts of variable size (de; **a**–**c**). Note that all postsynaptic target structures are GABA-negative, as indicated by the absence of *gold particles* in these structures. **d** Example of an axonal bouton establishing a synaptic contact with a small caliber GABAergic dendritic profile as indicated by the presence of *gold grains*. *Scale bars* in **a**–**d** are 0.5 μm

Strikingly, CR cells have a relatively high density of synaptic boutons along their axonal collaterals (Figs. 3c, 6). In the time window investigated here we found two- to threefold higher numbers compared to bouton counts of CR cells in *rat* (Fig. 6; Table 1; see Radnikow et al. 2002), CR cells in *reeler mice* (Radnikow et al., in preparation) and principal neurons taken from* rats *older in age (Lübke et al. 2000). We examined the synaptic contacts established by the axonal collaterals of biocytin-labeled CR cells in *CXCR4*-*EGFP mice* and their postsynaptic target structures in serial ultrathin sections through the entire axonal domain of CR cells (*n* = 2; Fig. 9). Axonal collaterals and synaptic boutons could be easily identified by the presence of the dark DAB-reaction product. Synaptic contacts can be characterized by the presence of synaptic vesicles of various sizes in the terminal, a clear synaptic cleft and sometimes a postsynaptic dense region (Fig. 9). Interestingly, in ~15 % of the cases investigated not all *en passant* boutons established synaptic contacts with a neighboring postsynaptic target dendrite or spine when followed in serial sections as also reported by Kubota et al. (2009) for synapses in the frontal cortex and Merchán-Peréz et al. (2009) using block face imaging of EM-preparations. To further identify the transmitter phenotype of the postsynaptic target structures, GABA postembedding immunogold labeling was carried out on some ultrathin sections.

The majority (~80 %) of synaptic boutons were found on dendritic shafts of variable size (Fig. 9a, b). A minor fraction (~10 %) of boutons established synaptic contacts with spine-like appendages (Fig. 9c). Furthermore, the majority of biocytin-labeled boutons investigated (*n* = 65) formed *en passant* synapses on non-GABAergic dendritic profiles suggesting that CR cells preferentially innervate non-GABAergic structures, presumably dendrites of other CR cells and/or terminal tuft dendrites of L2/3 and L5 pyramidal neurons. Infrequently, synaptic boutons were also identified on GABAergic dendritic segments (Fig. 9d) as identified by GABA post embedding immunohistochemistry. Thus, CR cell axons also establish synaptic contacts presumably with L1 GABAergic interneurons.

### GABAergic interneurons in layer 1 and their relation to CR cells

**Fig. 10:**
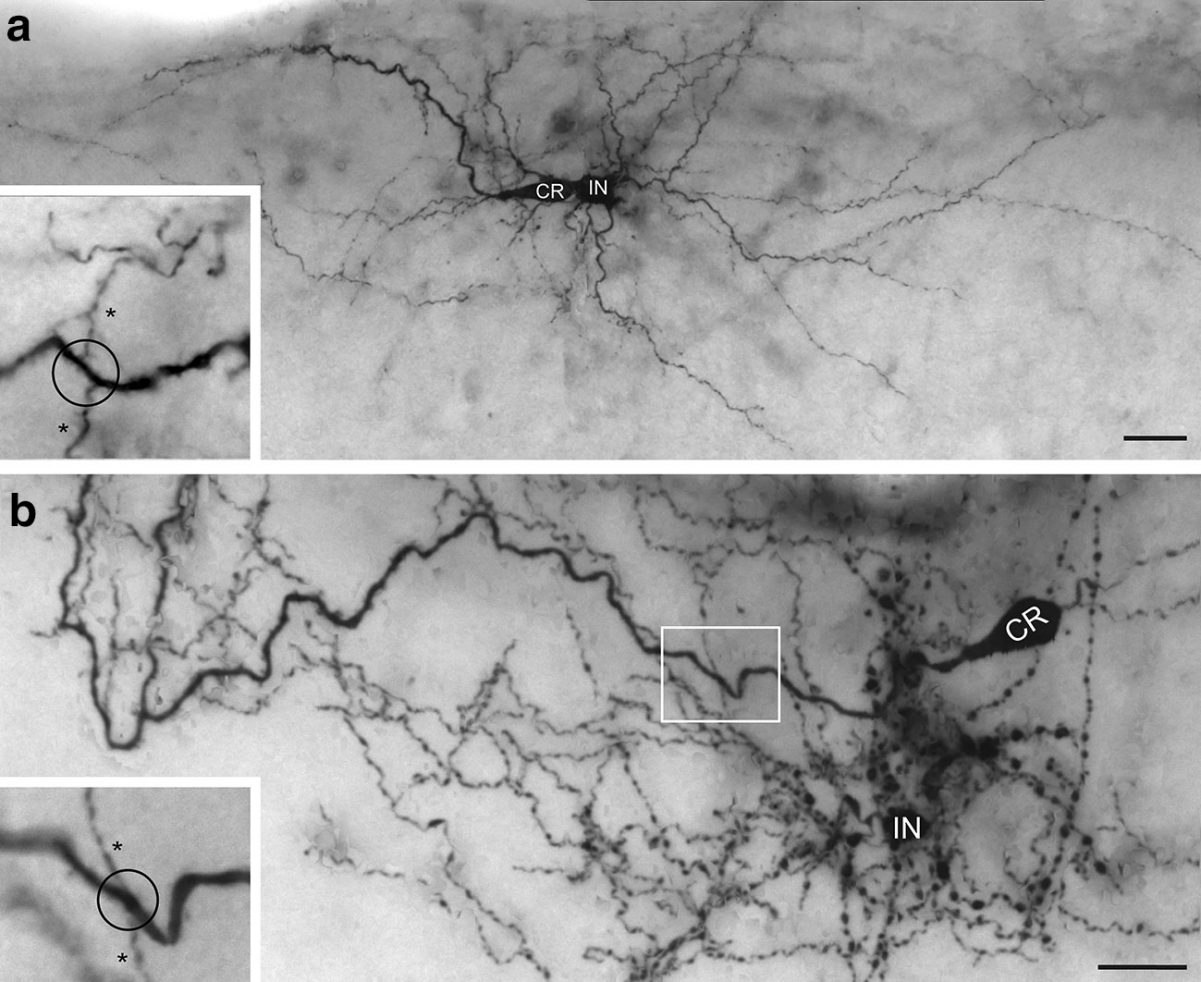
Light microscopy of CR cells and GABAergic interneurons in layer 1 of the neocortex. **a**, **b** Besides CR cells (*CR*), numerous GABAergic interneurons (*IN*) with different dendritic morphologies and axonal domains were found in layer 1. In both **a** and **b** one CR cell and one L1 interneuron is filled with biocytin. The interneuron in **a** displayed a multipolar elongated dendritic tree with a dense local axonal domain and individual long-range horizontal axonal collaterals confined to layer 1. The interneuron in **b** is of the neurogliaform type with local axonal collaterals and a high degree of collateralization and density of synaptic boutons. *Insets* in **a**, **b** High power light microscopy (framed area in **b**) suggests that the *en passant* axons (indicated by *asterisks*) of GABAergic interneurons establish synaptic contacts on proximal and distal dendrites (indicated by the *open circle*) of CR cells. *Scale bars* in **a**, **b** are 20 μm

**Fig. 11:**
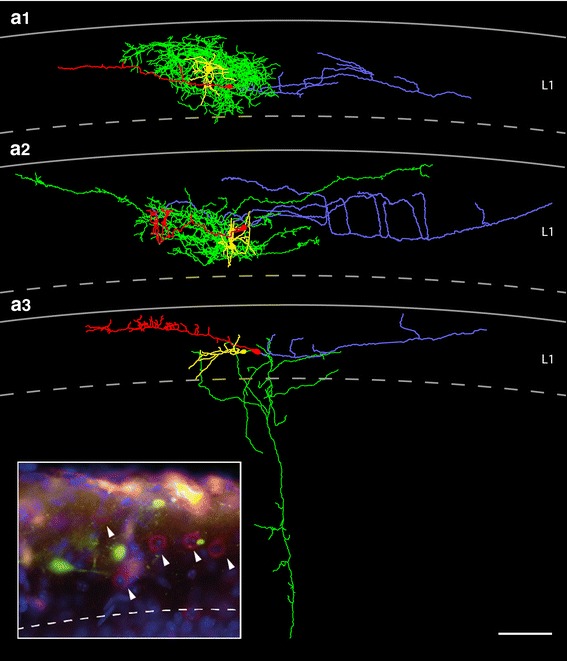
*Neurolucida* reconstructions of CR cells and GABAergic interneurons in layer 1 of the neocortex. **a1**–**a3** Three representative examples of CR cells and adjacent GABAergic interneurons. The somatodendritic domain of the CR cells is given in *red* that of the interneurons in *yellow*, the axonal domains in *blue* (CR cells) and *green* (interneurons). The interneurons depicted in **a** and **b** display a local and dense axonal domain nearly completely covering the dendritic domain of the CR cells whereas the interneuron in **c** possess an axon projecting deep into layer 5 with collaterals in layer 2/3 and layer 4. *Scale bar* in **a–c** is 100 μm. *Inset* GABAergic interneurons in layer 1 as revealed by Gad67-immunoreactivity (indicated by *arrowheads*) are intermingled with EGFP-labeled CR cells

**Fig. 12:**
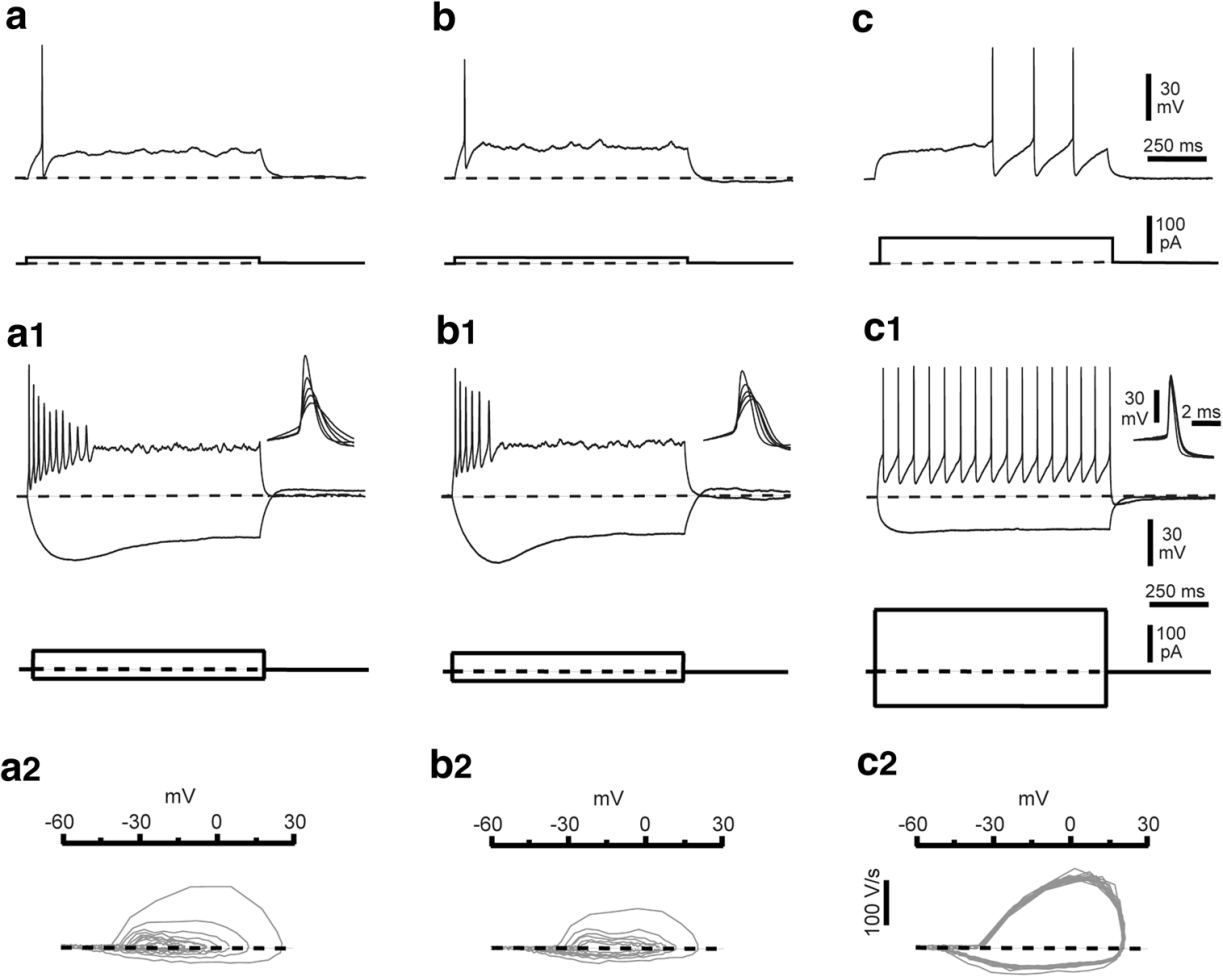
Firing patterns of CR cells compared to those of GABAergic interneurons in layer 1. **a**–**a2**, **b**–**b2** Voltage responses of typical (*left panels*) and ‘atypical’ (*middle panels*) CR cells to depolarizing and hyperpolarizing current pulses of 1 s duration; the membrane potential of both cells was held close to −60 mV. Panels **a** and **b** show the response to low amplitude positive current pulses, whereas panels **a1** and **b1** show firing patterns in response to stronger depolarizing and hyperpolarizing current pulses. **a2**, **b2** Phase plots of the trains of action potentials are displayed in the *middle* insets. Notice the typical progressive change of the action potentials. This is also shown in the insets where the first five action potentials have been adjusted so that the action potential thresholds coincide and superimposed. Trains of action potentials in CR cells were characterized by spike broadening and eventual depolarization block. Both CR cells show a typical ‘sag’ in the hyperpolarizing response, indicative of the presence of I_h_. **c**–**c2** Analogous to **a**–**a2** and **b**–**b2** but the recording was performed in a L1 GABAergic interneuron. Note the different size of the current steps used and the different shape of the action potentials and firing pattern and the marked difference in the phase plot of the trains of action potentials

In addition to CR cells a heterogeneous population of GABAergic interneurons was observed in layer 1 of acute slices using infrared video microscopy (Figs. 10, 11). GABAergic interneurons (see also Kubota et al. 2011; Wozny and Williams 2011; Jiang et al. 2013) at this time window (P7–P11) are densely packed in layer 1 intermingled with the population of CR cells and sometimes showed cluster-like arrangements around CR cells (Fig. 11 inset). GABAergic interneurons were never fluorescently labeled and can be clearly distinguished from CR cells by their dendritic arborization, axonal projection patterns after biocytin-filling and by their electrophysiological characteristics (Fig. 12). However, GABAergic interneurons investigated in *CXCR4*-*EGFP* *mice,* although our sample size is relatively small (*n* = 15), differ with respect to their dendritic configuration and axonal projection pattern. Besides neurogliaform-like interneurons with very thin, often beaded dendrites (Figs. 10b, 11a1), neurons with horizontally (Figs. 10a, 11a3) or more radially oriented dendrites (Fig. 11a2) were found. Thus, different types of GABAergic interneurons may be classified upon the axonal projection pattern: first, those with a local but dense axonal plexus confined to layer 1 (Fig. 11a1); a second type with a dense local plexus but with individual long-range horizontal collaterals also confined to layer 1 (Fig. 11a2) and third with two axonal domains, one local within layer 1 and a projection domain with a vertically oriented main axon giving rise to horizontally oriented axonal collaterals of different length terminating in layer 2/3 and layer L5 (Fig. 11a3).

We further made an attempt to first record and fill a CR cell and then a neighboring interneuron (Figs. 10, 11). The axonal domain of the GABAergic interneurons with an axon confined to layer 1 almost entirely covered the dendritic domain of the CR cells (Fig. 11a1, a2) which strongly suggest a synaptic interaction between these neurons. At the light microscopic level GABAergic interneurons were seen to establish putative synaptic contacts (close appositions between the axon and the target structure) at somata, proximal and distal dendrites of CR cells (Fig. 10 insets in a and b). Thus, it may be speculated that GABAergic interneurons in layer 1 are presynaptic partners of CR cells not only providing feed-forward inhibition but also control synchronous firing of CR cells (Soda et al. 2003) thereby modulating synaptic activity in these neurons.

### Electrophysiological characteristics

Membrane properties and firing pattern were not different in typical and ‘atypical’ CR cells and corresponded well to electrophysiological characteristics described previously for CR cells in younger animals in the neocortex (Fig. 12; see Kim et al. 1995; Zhou and Hablitz 1996a; Hestrin and Armstrong 1996; Kilb and Luhmann 2000; Radnikow et al. 2002) and in older *CXCR4*-*EGFP mice* in the hippocampus (Fig. 12, see also Marchionni et al. 2010) but different from GABAergic interneurons (Fig. 12c–c2) as described for *rats* by Hestrin and Armstrong (1996), Kubota and co-workers (2011), Wozny and Williams (2011) and Radnikow et al. (2002). The holding currents recorded at −60 mV immediately after breakthrough were −30 ± 6 pA vs. 23 ± 4 pA for typical (*n* = 14) and ‘atypical’ (*n* = 11 CR cells, respectively (*p* > 0.05). Both types of CR cells had a high input resistance (1.7 ± 0.3 GΩ in *n* = 14 typical CR cells vs. 1.8 ± 0.2 GΩ in *n* = 11 ‘atypical’ CR cells, *p* > 0.05). The firing pattern in response to depolarizing current steps showed a characteristic spike broadening with decreasing amplitude (Fig. 12a1, b1). A ‘sag', indicating the existence of an I_h_ current (Kilb and Luhmann 2000) was induced by hyperpolarizing current pulses (Fig. 12a1, b1). However, we found that this specific characteristic was particularly sensitive to run-down, probably reflecting the loss of critical intracellular components required to maintain I_h_ activity. At 29–31 °C and a membrane potential of –60 mV, the first action potentials of the train had a half width of 1.3 ± 0.1 ms in *n* = 12 typical and 1.1 ± 0.1 ms in *n* = 11 ‘atypical’ CR cells (*p* > 0.05). This is very different from *rat* CR cells, where the time course of the first action potentials in the train is already much slower and can be easily distinguished from that recorded in GABAergic interneurons (Radnikow et al. 2002; Kubota et al. 2011; Wozny and Williams 2011). We could not find any differences in the action potential half width of either typical or ‘atypical’ CR cells when compared with layer 1 GABAergic interneurons (Fig. 12c–c2, 1.1 ± 0.2 ms, *p* > 0.05). This finding suggests a *mouse-rat* species-dependent difference in the density or expression of voltage-gated channels contributing to the shape of CR cell action potentials. Nevertheless, the firing pattern and action potential properties of CR cells were usually quite distinct from that observed in L1 GABAergic interneurons (compare Fig. 12a–a2, b–b2 with c–c2).

## Discussion

### The CXCR4-EGFP mouse as a tool to identify CR cells in neocortical layer 1

The results of our work validate the *CXCR4*-*EGFP mouse* as a tool to facilitate the identification of CR cells in developing layer 1. In fact, within this layer, we never encountered EGFP-expressing neurons other than CR cells (see “Results”). This observation is consistent with the critical role of *CXCR4* activity in setting the laminar position of CR cells in the brain (Paredes et al. 2006). It is also interesting to note that we were unable to visually find neurons with CR cell morphology and CR cell electrophysiological properties that were not EGFP-labeled, which strongly suggest that *CXCR4* is expressed by CR cells of different ontogenetic origin (Bielle et al. 2005).

### Fate of CR cells in the rat neocortex

The fate of CR cells is still subject of ongoing controversy being attributed to species differences or either to selective cell death, dilution in the developing neocortex or differentiation into other cortical cell types (Edmunds and Parnavelas 1982; Parnavelas and Edmunds 1983; Derer and Derer 1990, 1992; Zecevic and Rakic 2001; for review see Marín-Padilla 1998; Mienville 1999; Meyer et al. 1999, 2000). Recently, in vivo two-photon microscopy of *Ebf2*-*GFP mice* (Chowdhury et al. 2010) has elegantly shown that the density of CR neurons is relatively stable between P3 and P4 to P7 but then started to progressively decline until P14 and finally stabilizes at low values somewhat after P20. In the end, only <3.5 % of CR neurons that are present at P3–P7 can be found after the fourth postnatal week. Interestingly, the rate of disappearance of CR cells is not constant throughout postnatal development; CR cell loss was fastest at P11–P13 (56.6 ± 5.5 %), compared to P7–P9 (73.4 ± 3.3 %) or P18–P22. In addition, long-lasting time-lapse video microscopy revealed that postnatal CR cells undergo dramatic morphological transformations, but their gradual disappearance from the cortex is due to apoptotic death during the second postnatal week (Chowdhury et al. 2010, see this paper). These findings are in line with this study where we can demonstrate that CR cells in *CXCR4*-*EGFP mice* had their highest density between P3 and P7 and then decline dramatically to be nearly abolished at P14 as indicated by the increase of Caspase-3-positive CR cells and signs of neuronal degeneration starting around P8. As already mentioned CR cells in the hippocampal formation are still found at very high densities when neocortical CR cells already have disappeared completely. This may reflect a differential time in birth between neocortical and hippocampal CR cells.

### Synaptic input to and output from CR cells in CXCR4-EGFP mice

Rather little is known about the input–output relations of CR cells in an early cortical network. No studies in *mice* are presently available that provide detailed information about the nature, extent, or origin of synaptic inputs to CR cells. Ultrastructural data in different species, so far, suggested that early postnatal CR cells either receive no synaptic input (König and Marty 1981) or only a few input synapses (Edmunds and Parnavelas 1982; Parnavelas and Edmunds 1983; Derer and Derer 1990, 1992). Furthermore, the fate of synaptic inputs during development is still a matter of debate; their number may either increase (Parnavelas and Edmunds 1983) or decrease (Derer and Derer 1990, 1992). In line with Edmunds and Parnavelas (1982) and Radnikow et al. (2002), we found that in P7–P11 *CXCR4*-*EGFP mice*, CR cells receive relatively dense synaptic input not only on the somatic region but also on proximal as well as distal dendrites and spine-like, filopodial protrusions. Both non-GABAergic and GABAergic input synapses were present at all ages investigated although the majority of inputs are non-GABAergic (see “Results”).

This is somewhat surprising, as the vast majority, if not all, of the spontaneous synaptic events recorded from CR cells appear to be sensitive to GABA_A_ receptor antagonists. Nevertheless, evoked glutamatergic synaptic responses characterized as pure NMDA receptor mediated PSPs or PSCs, (but no AMPA/kainate receptor mediated responses) have also been observed in morphologically identified *rat* CR cells (Radnikow et al. 2002). Similarly, only whole-cell NMDA responses were present in *mouse* CR cells while AMPA receptor responses were present in *human* CR cells (Lu et al. 2001). In addition, calcium imaging of CR cells showed a much weaker response to AMPA than to NMDA (Schwartz et al. 1998). However, in young CR cells (P0–P4) the occurrence of both non-NMDA and NMDA PSCs has been reported (Kim et al. 1995). In summary, our structural data highlight an important role for a putative glutamatergic drive of CR cells, despite the apparent lack of spontaneous glutamatergic events detected in electrophysiological recordings in vitro (Kilb and Luhmann 2001; Cosgrove and Maccaferri 2012). Besides the inputs described here, CR cells may receive synaptic inputs using other neurotransmitters. Serotonergic inputs from the raphe nuclei or noradrenergic inputs from locus coeruleus reach the cortex early during development (Parnavelas et al. 1988) and may establish synaptic contacts with CR cells. This idea is further supported by calcium imaging (Schwartz et al. 1998) suggesting the presence of β-adrenergic receptors on CR cells. Furthermore, α_2A_-adrenergic receptors have been detected in *monkey* CR cells (Wang and Lidow 1997).

CR cells establish functional output synapses, one pre-requisite for an active role in an early cortical network. It has been previously suggested that CR cell axons form synaptic contacts with pyramidal cell dendrites (Derer and Derer 1990; Marín-Padilla 1998). This was first demonstrated and confirmed by an electron microscopic analysis of biocytin-filled CR cell axons in* rat* (Radnikow et al. 2002). In *CXCR4*-*EGFP mice* CR cells establish *en passant* asymmetric synaptic contacts preferentially on dendritic shafts or spines of non-GABAergic neurons. The terminal tuft dendrites of neocortical layer 2/3 and 5 pyramidal neurons are the most frequent non-GABAergic structures in layer 1 forming a dense dendritic network (Jiang et al. 2013; for review see Marín-Padilla 1998). Thus, these neurons are most likely the main target structures of CR cells. Another source of target neurons for CR cell axons are other CR cells and layer 1 GABAergic interneurons. However, this has to be investigated in more detail. The ultimate experimental proof could be paired recordings from CR cells and their postsynaptic target neurons.

### Functional relevance of the long-range horizontal axonal projection and the high density of synaptic boutons of CR cells

Our results suggest that CR cells in *CXCR4*-*EGFP mice* are active elements in an early neuronal network by their long-range horizontal axons and density of boutons, by receiving relatively dense synaptic input and by providing functional output. One may speculate that the existence of a rather extensive neuronal network of CR cells in layer 1 (Fig. 4) point to a role of CR cells to integrate cortical signal flow originating from neurons in the underlying cortical layers or in layer 1 itself over a wide area of cortical surface already in an immature neocortex. Strikingly, CR cells in *CXCR4*-*EGFP mice* were characterized by long-range horizontal axons with a field span of ~1.7 mm within a time window where excitatory neurons in the underlying cortical layers are still in the process of maturation with respect to their dendritic arborization, axonal projection and their input–output properties. These long-range horizontal axons are comparable with estimates for CR cells in *rat* (Radnikow et al. 2002) but in marked contrast to previous studies in *mouse, rat* and *humans* where the axonal field span never exceeded 500 μm (Derer and Derer 1990; Marín-Padilla 1990; Hestrin and Armstrong 1996; Aguiló et al. 1999; Kilb and Luhmann 2001).

Such widespread axons together with the relatively high number of synaptic boutons and synaptic contacts preferentially established with non-GABAergic profiles, presumably other CR cells and terminal tuft dendrites of pyramidal neurons suggest a role for CR cells in an early cortical network which may represent a ‘pre-requisite’ for the establishment of the cortical column. One might speculate that long-range horizontal axons forming such a dense horizontal network throughout layer 1 (Fig. 4) very early in development could ‘anchor’ terminal tuft dendrites of pyramidal neurons by a dense network of synaptic contacts in layer 1 as shown in this study. This may contribute to the positioning and orientation of pyramidal neurons leading to the establishment of individual cluster-like arrangements or ‘bundles’ of apical dendrites of L2/3 and L5 pyramidal neurons into so-called ‘dendrons’. These ‘dendrons’ have been proposed to contribute to so-called ‘minicolumns’ that comprise small clusters of neurons in the cortical column (Fleischhauer et al. 1972; Escobar et al. 1986; DeFelipe 2005).

Furthermore, it has been shown that terminal tuft dendrites of L2/3 and L5 pyramidal neurons can span several cortical columns. Terminal tuft dendrites are the Ca^2+^ spike initiation zone that interacts with the Na^+^ action potential initiation zone in the axon of the same neurons. This interaction may be responsible for regenerative potentials critical for the integration and amplification of sensory and modulatory inputs (Larkum et al. 1999; Larkum and Zhu 2002; Jiang et al. 2013) even early in the development. This may result in an integrated synaptic activity of developing pyramidal cells by the activation of Ca^2+^ spikes in pyramidal cells across columns, thereby contributing to the establishment of cortical domains. CR cells in layer 1 may actively trigger the induction, maintenance and modulation of this Ca^2+^ spikes by their long-range horizontal axons, the high density of output synapses and their network activity. The activity of CR cells themselves may be controlled by L1 GABAergic interneurons (Wozny and Williams 2011; Jiang et al. 2013; reviewed by Le Magueresse and Monyer 2013) that may modulate the excitatory drive by feed-back and feed-forward inhibition preventing over-excitation of the network.

### Dye-coupling in the neocortex

Besides synaptic contacts the phenomenon of dye-coupling is a frequent observation between the same class and/or different types of neurons in the immature neocortex (LoTurco and Kriegstein 1991; Yuste et al. 1992; Peinado et al. 1993b; Rörig et al. 1996; for review see Peinado et al. 1993a). It has been proposed that dye-coupling is an indicator for the presence of gap junctions (Gutnick and Prince 1981; Connors et al. 1983; LoTurco and Kriegstein 1991; Yuste et al. 1992; Peinado et al. 1993b; Rörig et al. 1996). Dye-coupling has been suggested to be functionally relevant for cell–cell communication in an early cortical network since the formation of chemical synapses between individual neuronal connections occurs around P6. Interestingly, and in contrast to a study of CR cells in *rat* neocortex (Radnikow et al. 2002), dye-coupling was not observed in *CXCR4*-*EGFP mice*. The recording and biocytin-labeling of individual CR cells in *CXCR4*-*EGFP mice* always resulted in the detection of a single CR cell without even a weak labeling of neurons in the surrounding neuropil. The staining was always very clear without any extracellular dye deposits in the surrounding tissue. The lack of dye-coupling may be explained by the fact that the cell membrane of EGFP-labeled CR cells in *CXCR4*-*EGFP mice* are somehow less or non-permeable for the diffusion of biocytin via gap junctions. However, we cannot rule out the possibility of dye-coupling since our recordings were made in a time window (P7–P11) where the incidence of dye-coupling decreases dramatically during postnatal development, being almost non-existent after the second postnatal week (Connors et al. 1983; LoTurco and Kriegstein 1991; Kim et al. 1995; Rörig et al. 1996).

Strikingly, biocytin-labeling of an individual CR cell sometimes resulted (in ~30 % of the cases investigated) in the co-labeling of astrocytes with an end feet contacting the pial surface (Fig. 3d). At the light microscopic level, the stem dendrite of the CR cell sometimes crossed the arborization of the labeled astrocytes when located close to the recorded neuron. In addition, we found astrocytes relatively far away (~50–70 μm) from the recorded neurons where the ‘dendritic tree’ seems to be contacted by synapses of the CR cells (Fig. 3d). Thus, it may be speculated that CR cells establish both gap junctional and synaptic coupling with these astrocytes. We found synaptic coupling between astrocytes and synaptic boutons near the pial surface (see Fig. 3c3) as confirmed by electron microscopy but infrequently. It has been shown that astrocytes actively shape the dynamics of neurons and neuronal ensembles by affecting several aspects critical to neuronal function, such as regulating structural and functional synaptic plasticity, modulating neuronal excitability, and maintaining extracellular ion balance (Wade et al. 2011; Volman et al. 2012). Recently, it has been demonstrated that astrocytic signaling controls and modulates spike-time dependent depression at neocortical L2/3 and L4 synapses (Min and Nevian, 2012). It may be hypothesized that the astrocyte-CR cell interaction may partially contribute to the regulation and modulation of the activity of CR cells thereby shaping the network properties of layer 1.

### A possible functional role of CR cells

CR cells secrete *reelin*, an extracellular matrix glycoprotein that is essential for the radial migration of excitatory neurons from the ventricular zone into the cortical plate (D’Arcangelo et al. 1995, 1997, reviewed by Tissir and Goffinet 2003). During the early stages of corticogenesis, CR cells are the only source of *reelin*, while *reelin*-positive GABAergic interneurons appear later in the development (Alcantara et al. 1998; Meyer and Goffinet, 1998). CR cells release high amounts of *reelin* into the extracellular matrix where it is taken up by radial glia and radially migrating neurons that express the *reelin* receptors ApoER2 and VLDLR and the intracellular adapter protein disabled 1 (Dab1; Rice et al. 1998; D’Arcangelo et al. 1999; Benhayon et al. 2003). The integrity of this signaling pathway is essential for correct positioning of cortical-plate neurons; disruption of any of its components leads to a failure of radial migration (Trommsdorff et al. 1999; Howell et al. 2000; Kuo et al. 2005). In rodents, mutations of the genes involved in the *reelin*-Dab1 pathway lead to a common ‘*reeler*-like’ phenotype, in which the cortical layers are grossly inverted and layer 1 is missing.

In addition, we demonstrate that CR cells receive dense non-GABAergic and to a lesser extent GABAergic synaptic input and in turn provide synaptic output not only to other CR cells and GABAergic interneurons but also to pyramidal neurons of the underlying cortical layers. Thus, CR cells are an integrative element of an early cortical network not only but preferentially in layer 1. CR cells form a dense axonal network with their long-range horizontal axons that establish synaptic contacts over a wide range of cortical surface (Fig. 4). The interaction between CR cells and pyramidal cells of the underlying cortical layers may constitute an interface network until the latter receive their final afferents in the mature neocortex (Jiang et al. 2013). With respect to neocortical organization, CR cells may play a role reminiscent of that of ‘subplate’ neurons which provide a scaffold for thalamocortical afferents (McConnell et al. 1989; Friauf et al. 1990; Goodman and Shatz 1993) with the difference that ‘subplate’ neurons are transient targets of these afferents while CR cells provide synaptic input to other CR cells, GABAergic interneurons and pyramidal neurons in the underlying cortical layers. One may speculate that this scaffold is required during early stages of sensory map formation.
